# Primary Diffuse Large B-Cell Lymphoma of the Urinary Bladder: A Case Report and Literature Review

**DOI:** 10.7759/cureus.90579

**Published:** 2025-08-20

**Authors:** Sebastian Salinas Mendoza, Victoria Eugenia Lora Marquez, Juan Carlos Velez Roman

**Affiliations:** 1 Internal Medicine, Hospital Serena del Mar, Cartagena, COL; 2 Hematology, Hospital Serena del Mar, Cartagena, COL; 3 Urology, Hospital Serena del Mar, Cartagena, COL

**Keywords:** bladder tumor, gross haematuria, large b cell lymphoma, primary bladder lymphoma, transurethral resection of bladder tumor (turbt)

## Abstract

Primary urinary bladder lymphoma is a very rare disease. Hereby, we report a 53-year-old male patient who presented with sudden macroscopic hematuria. The patient had a relevant previous history of follicular non-Hodgkin lymphoma (NHL) treated initially with chemotherapy CHOP scheme and then with bendamustine-obinutuzumab, with a PET scan at the end of the treatment showing a complete response. Abdomen-pelvic tomography revealed a diffuse and irregular thickening of the bladder. In the cystoscopy, we observed multiple solid-appearing tumor lesions throughout the bladder. A biopsy was taken, with a report of diffuse high-grade large B-cell NHL. Lymphomas are a rare cause of malignant bladder tumors, with DLBCL (diffuse large B-cell lymphoma) being the least common. Given its rare presentation, there is still no standardization of the treatment; a few reports and series have been published, offering surgical, chemotherapy, or radiotherapy-guided treatment, or frequently a combination of those methods. We are still lacking more information about this condition, aiming to choose the alternative treatment.

## Introduction

Non-Hodgkin lymphoma (NHL) is the most common hematologic malignancy, with over 40 subtypes. Of these, 80-95% originate from the B-cell lineage, while the remainder derive from T lymphocytes or NK cells. Follicular lymphoma and diffuse large B-cell lymphoma (DLBCL) are the most frequent subtypes [1,2]. Up to 40% of lymphomas may present with extranodal involvement, primarily affecting the stomach, central nervous system, connective tissue, bones, breast, testes, and skin. Primary bladder lymphomas are exceptionally rare, accounting for only 0.2% of all extranodal lymphomas and less than 1% of bladder tumors [3]. Most primary bladder lymphomas are low-grade malignancies, predominantly marginal zone lymphomas or mucosa-associated lymphoid tissue (MALT) lymphomas [4,5]. The frequency of presentation varies according to each report [6,7]. Treatment selection (chemotherapy, surgery, and/or radiation) depends on the lymphoma subtype. However, due to the rarity of bladder lymphomas, no standardized treatment protocol exists. This study reports a case of primary bladder DLBCL and reviews the literature to synthesize the clinical characteristics, treatment approaches, and patient outcomes for this uncommon malignancy.

## Case presentation

**Figure 1:**
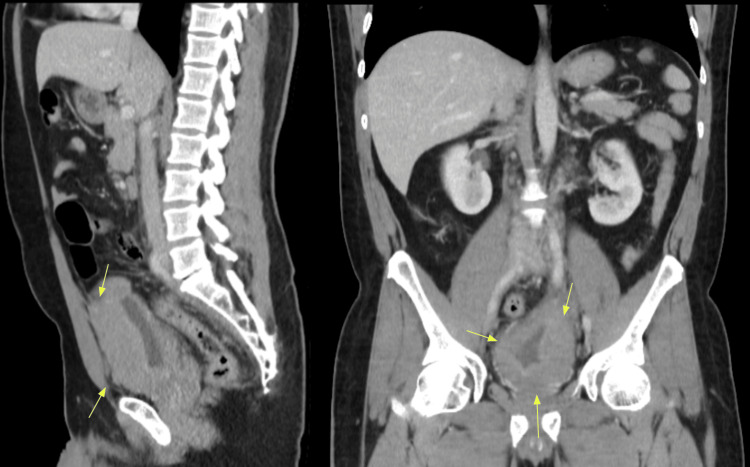
Contrast-enhanced abdominal and pelvic computed tomography. Sagittal (left) and coronal (right) views demonstrating diffuse irregular bladder wall thickening.

**Figure 2:**
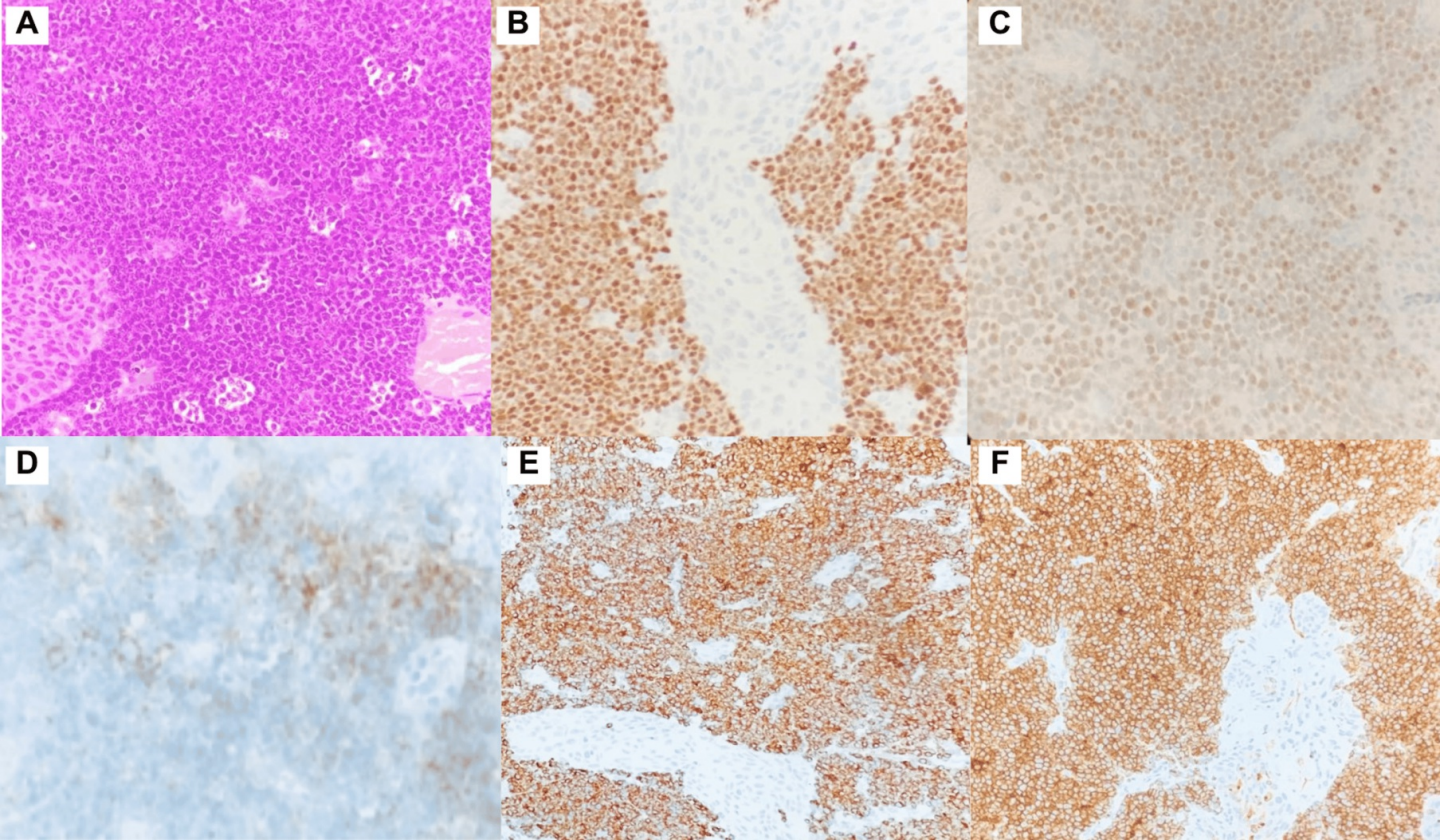
Hematoxylin and eosin (H&E) staining and immunohistochemistry. A: Intermediate-sized lymphoid cells with scant cytoplasm, nucleus showing frequent mitoses and blastoid chromatin. B: KI-67 proliferation index of 100%. C: C-MYC 80%. D: Focal and weakly positive CD20 expression. E: Nuclear BSAP positivity. F: CD10-positive staining.

A 53-year-old man with a history of follicular NHL initially treated with the CHOP protocol (due to rituximab intolerance) presented with early relapse. He subsequently achieved complete metabolic remission (PET-CT confirmed) following obinutuzumab-bendamustine therapy. One month post-treatment completion, the patient presented to the emergency department with macroscopic hematuria, dysuria, and vesical tenesmus. Contrast-enhanced CT of the thorax, abdomen, and pelvis (Figure 1) revealed diffuse irregular bladder wall thickening without metastatic lesions. Cystoscopy demonstrated multiple solid tumor-like lesions predominantly in the left lateral wall and bladder floor. Histopathological analysis of biopsy specimens showed extensive bladder involvement by a high-grade B-cell lymphoma with a starry-sky pattern (Figures 2a, 2b). Immunohistochemistry was positive for CD20, CD10, BCL2, MUM1, and MYC and showed 100% KI67 proliferation index (Figures 2c-2f). The findings were consistent with high-grade B-cell lymphoma. Bone marrow aspiration and biopsy were performed, showing no evidence of marrow involvement.

During hospitalization, the patient developed recurrent bleeding with consequent anemia requiring transfusion support, along with obstructive acute kidney injury secondary to hematuria. A repeat cystoscopy was performed for clot evacuation. The hematology team reviewed the histopathological findings and initiated treatment with the R-DA-EPOCH protocol. The patient succumbed to infectious complications following the second treatment cycle.

## Discussion

We conducted a scoping review of observational studies, including cohort studies, case-control studies, case reports, and case series on primary bladder lymphomas. Our search strategy utilized the MeSH, Emtree, and DeCS terms: "Bladder," "urinary tract," "lymphoma," and "non-Hodgkin lymphoma" across major databases: MEDLINE (via PubMed), Cochrane Library, EMBASE, and LILACS.

We examined reference lists of identified studies and performed gray literature searches through Google and Google Scholar. Exclusion criteria comprised: pediatric studies, primary lymphomas of the ureter/urethra/kidney/prostate, and cases with suspected secondary infiltration. Given the rarity of this condition, we anticipated that no clinical trials would be available. No language or date restrictions were applied. All databases were searched through May 30, 2025. The complete search strategy is detailed in Appendix 1.

Results

We identified 69 publications (56 case reports and 13 case series) comprising 125 patients with primary bladder lymphoma, most frequently reported in the United States, England, and Japan. Table 1 provides an overview of the key characteristics of reported cases. A comprehensive description of these cases is presented in Table 2, in the Appendices. The median age was 68 years (IQR 57-76) with a female predominance (ratio 1.78:1). Macroscopic hematuria was the most common presentation, though 12% of cases initially received empirical UTI treatment. Cystoscopy revealed solitary lesions in 58% of documented cases, while imaging (ultrasound, CT, MRI, or urography) typically showed bladder masses (64%), wall thickening (29%), or hydronephrosis/ureterohydronephrosis (18%). Low-grade lymphomas (64.7% of cases) were predominantly MALT type, while DLBCL accounted for most high-grade cases; 89% were stage IE/A without marrow involvement. Treatment approaches varied (surgery, chemotherapy, radiotherapy, and/or antibiotics), with higher remission rates in low-grade (84%) versus high-grade (69%) lymphomas (median follow-up 36 vs. 16 months, respectively). Seven patients received antibiotics (UTI-directed or anti-H. pylori) [8-14]. Received H pylori treatment with failure, secondary radiotherapy induced remission [15].

**Table 1:** Characteristics of reported cases of primary bladder lymphoma up to May 2025. MALT: mucosa-associated lymphoid tissue; DLBCL: diffuse large B-cell lymphoma; R-CHOP: rituximab-cyclophosphamide-doxorubicin-vincristine-prednisolone; TURBT: transurethral resection of bladder tumor, with antibiotics including TMP (trimethoprim), doxycycline, and H. pylori triple therapy (clarithromycin/amoxicillin/PPI). Source: [3,4,8-75]

Cases		N 125
Age (median, IQR)		68 (57–76)
Sex (%)		
	Male	45 (36)
	Female	80 (64)
Study (%)		
	Cases series	68 (54.4)
	Case reports	57 (45.6)
Country (%)		
	United States	38 (30.4)
	England	22 (17.6)
	Japan	18 (14.4)
	United Kingdom	12 (9.6)
	China	5 (4.0)
Symptom (%)		
	Gross hematuria	48 (28.4)
	Pain urination	25 (14.8)
	Urinary infection	21 (12.4)
	Frequency	20 (11.8)
	Low back pain	6 (3.6)
	Incidental	6 (3.6)
	Urgency	7 (4.1)
Images (%)		
	CT	40 (71.4)
	US	8 (14.2)
	MRI	4 (7.14)
Findings (%)	Tumor	27 (36.4)
	Wall thickening	20 (27.03)
	Hydronephrosis	16 (21.62)
	Filling defect	4 (5.41)
Cystoscopy (%)		
	Focal lesion	34 (53.1)
	Wall thickening	6 (9.38)
	Erythematous Mucosa	6 (9.38)
	Bulging	3 (4.69)
Localization (%)		
	Right wall	9 (22.5)
	Left wall	6 (15.0)
	Posterior wall	7 (17.5)
	Trigone	4 (10.0)
Grade (%)		
	High grade	42 (35.3)
	Low grade	77 (54.7)
Histology (%)		
	MALT	63 (51.22)
	DLBCL	31 (25.2)
	Follicular	5 (4.0)
State (%)		
	I	46 (71.8)
	II	5 (7.8)
	III	1 (1.5)
	IV	12 (18.7)
Outcome (%)		
	Remission	83 (72.8)
	Dead	15 (13.2)
Relapse (%)		
	Yes	10 (8.8)
	No	36 (31.9)
Treatment (%)		
	Chemotherapy	33 (26.4)
	Radiotherapy	24 (19.2)
	Surgery	16 (12.8)
	Chemotherapy + Surgery	15 (12.0)
	Chemotherapy + Radiotherapy	5 (4.0)
	Surgery + Radiotherapy	12 (9.6)
	Chemotherapy + Surgery + Radiotherapy	5 (4.0)
Chemotherapy schemes (%)		
	R CHOP	11 (9.6)
	CHOP	7 (6.1)
	Rituximab	5 (4.4)
	Antibiotics	8 (7)
Chemotherapy cycles (IQR)		6 (4 - 6)
Radiotherapy (%)		50 (43.9)
	Yes	46 (44.8)
	No	23 (18.4)
	Median Gy (IQR)	35 (30-35.25)
	Sessions median (IQR)	20 (17 - 20)
Surgery (%)		
	Yes	48 (78.6)
	No	13 (21.3)
Type of surgery (%)		
	TURBT	35 (71.4)
	Cystectomy	4 (8.16)
	Partial cystectomy	3 (6.12)
Outcome (%)		
	Remission	92 (78.6)
	Dead	16 (13.6)
Outcomes MALT (%)		
	Remission	51 (86.4)
	Dead	4 (6.7)
Outcomes DLBCL		
	Remission	19 (67.86)
	Dead	6 (21.4)
Median remission (IQR)		
	High grade	16 (6-54)
	Low grade	36 (12-60)
Relapse (%)		10 (8.62​​)

In our review, 15 low-grade lymphoma cases were treated with chemotherapy alone with different regimens: rituximab monotherapy, R-CHOP, CHOP, or chlorambucil/prednisolone/vincristine [7,11,16-26]. Of these, eight achieved complete remission [7,11,16,18,20-24,26], three showed partial response [19,24,25], and one death was recorded [11]. Among 15 cases treated with radiotherapy alone [11,20,27-32], the common dosages and sessions were 35-50 Gy in 17-20 fractions, 12 attained complete remission [11,20,27-32], and two fatalities, one at 36-month follow-up [20] and another case lost to documentation [31]. Surgical approaches were done for nine cases: six transurethral bladder resections (TURBT) [4,7,11,20,31,33-36], two cystectomies [20,31], and one diathermy [11]. All patients achieved complete remission, though there was one case of relapse at 12 months [7]. Combined therapies demonstrated high effectiveness: there were three cases of chemotherapy plus radiotherapy [24,31,37], and nine cases of chemotherapy plus surgery [3,20,38-43]. Both strategies achieved 100% complete remission rates. Similarly, the radiotherapy-surgery combination (14 cases) produced complete remissions in all patients [3,10,15,20,40,44-48], with one relapse that subsequently responded to salvage radiotherapy and anti-H. pylori treatment [15]. There were four cases of combined surgery, radiotherapy, and chemotherapy with remission observed in all [10,20,40]. Notable outcomes emerged in seven antibiotic-treated cases: three combined with TURBT [11-13], and four as monotherapy [8,9,11,14], all achieving complete remission and remaining relapse-free during follow-up (6-36 months). We identified cases in which patients did not receive treatment. One involved a patient who defaulted on follow-up but remained alive with the disease for three years [49]. Another was followed for three years without therapy; the patient later returned with obstructive urinary symptoms, which improved following a prostatic resection [50]. A third patient was observed for one year without treatment and ultimately died from a myocardial infarction [31].

Our review identified 20 high-grade lymphomas [3,20,21,24,26,37,49,51-64]. Of these, 16 patients were treated with chemotherapy alone-using regimens such as CHOP, R-CHOP, or MACOP-B-resulting in remission in 10 cases [11,21,37,51,54,57,61,64]. One of these experienced relapse at five months, successfully salvaged with chemo-radiotherapy [37]. Three patients died [11,24,26], and two had disease progressions [60,63]. Radiotherapy alone was employed in seven cases, achieving remission in four and resulting in one death [20,49]. Surgical monotherapy, applied in three cases-partial cystectomy [3,20], and TURBT [58], led to remission in all, though one patient died following relapse at 36 months [3]. Three cases involved combined chemotherapy and radiotherapy, with remission observed in all except one [20,46,49]. Four cases received a combination of chemotherapy and surgery, with remission observed in all except one [3,11,20,59]. One patient received triple-modality therapy (chemotherapy, radiotherapy, and surgery), which resulted in complete remission [46] as shown in Appendix 2.

Our review identified five cases of follicular lymphoma [20], two of which were treated with combined chemotherapy, radiotherapy, and surgical interventions (superior vaginectomy and lesion fulguration), both resulting in remission. One of them was treated with radiotherapy and TURBT, who died nine years after. One case of Burkitt lymphoma was fatal during induction chemotherapy [26]. A lymphoblastic lymphoma case achieved remission following chemotherapy [37]. We found three cases of lymphocytic lymphoma [23,29,50]. One patient with anaplastic lymphoma attained remission after undergoing chemosurgical treatment [11]. Lastly, two cases of T-cell lymphoma resulted in fatal outcomes [65,66]. 

Primary bladder lymphomas are rare malignancies that may exhibit any histological subtype, though MALT lymphomas followed by DLBCL are most frequently reported. The most common clinical manifestations include hematuria, dysuria, and urinary frequency. Imaging typically reveals solitary or multiple masses, with only 10% of cases presenting as irregular bladder wall thickening [37,45,60].

The retrospective cohort study [6], by Lontos (1983-2013), identified 1,264 patients with primary urinary tract lymphoma, estimating an annual bladder lymphoma incidence of 0.3 cases per 1,000,000 individuals. Patients had a mean age of 76 years with female predominance (58.7%). Histological distribution showed DLBCL (60.3%), MALT (22.7%), and follicular lymphoma (7.1%), similar to the findings in Seki’s review [60]. Contrary to those findings, in this review, we found MALT as the main type of lymphoma, followed by DLCBL, similar to previous publications [7]. We found gross hematuria, painful urination, and urinary tract infection as the principal symptoms; these findings are similar to those reports in Seki reviews, most of which are indistinguishable from urinary tract infection [60]. We suggest that in patients with recurrent UTI associated with hematuria, the diagnosis of primary bladder lymphoma should be considered. 

In the biggest retrospective cohort study [6], Lontos reports primary treatments based on surgery alone (43.9%), non-surgical/radiation approaches (32%), radiation alone (4.9%), and combined surgery-radiation (17%), with an overall five-year survival rate of 63%. This is also different from our observation, where patients were principally treated with chemotherapy (26%), followed by radiotherapy alone (19.2%). We believe this difference could be seen because of the frequency of the lymphomas' presentations.

MALT lymphoma emerged as the most prevalent subtype of primary bladder lymphoma, accounting for 51% of cases in this review. A variety of treatment modalities were reported, observation, antibiotic, chemotherapy, radiotherapy, surgery, and combination approaches, with generally favorable remission rates. Regardless of the approach chosen, high remission rates were consistently observed. The Richard Tsang study evaluated radiotherapy response in 69 MALT lymphoma patients, including three with primary bladder involvement, demonstrating a 76% disease-free survival and 96% overall survival rate. Based on these outcomes, the authors propose low-dose radiotherapy (25-30 Gy in 15-20 fractions) as an effective treatment option for stage I-II MALT lymphomas [7,67]. Therefore, we suggest that systemic therapy be reserved as a last resort, given the promising outcomes associated with more conservative interventions.

High-grade lymphomas account for 20% to 40% of primary bladder lymphoma cases [60]. Chemotherapy, primarily the R-CHOP regimen, constitutes the mainstay treatment for primary bladder DLBCL, with untreated patients facing twice the mortality risk. Overall, patients with urinary tract DLBCL demonstrate an ambiguous prognosis, as survival outcomes remain incompletely characterized due to limited case volumes and heterogeneous treatment approaches [5]. In our review, DLBCL represented 24% of cases, with various treatment approaches employed (chemotherapy, radiotherapy, or TURBT), we found a 67% response rate and median remission duration of 16 months. Given the potential for undetected systemic disease, we suggest that a combination therapy including chemotherapy remains the treatment of choice, with R-CHOP being the most common scheme used. While 60% of treated cases achieve remission, the rarity of this condition necessitates further studies to better characterize the benefits of chemoimmunotherapy. 

We have presented a case of DLBCL of the bladder. In this instance, the patient died from an infectious complication likely related to the intensity of the systemic chemotherapy regimen. While the prognosis of bladder DLBCL remains uncertain due to its rarity and clinical heterogeneity, the literature suggests that not all patients with bladder lymphoma require systemic treatment. We believe that individuals with reduced fitness may benefit from less intensive therapeutic approaches, including localized radiotherapy and/or surgical resection, tailored to disease extent and patient tolerance.

## Conclusions

Despite this representing the largest case compilation to date, several limitations warrant consideration: potential underreporting of bladder-specific involvement may have led to case exclusion, the retrospective design introduces inherent selection biases, and heterogeneous treatment approaches preclude definitive therapeutic conclusions. These constraints emphasize the need for prospective, standardized studies to validate our findings. Primary bladder lymphomas represent rare clinical entities typically exhibiting indolent behavior. As the largest case compilation published to date, this review synthesizes available evidence despite persistent heterogeneity in clinical presentation and treatment responses. By consolidating historical experiences, we aim to provide clinicians with a consolidated evidence base to guide therapeutic decision-making for this uncommon malignancy.

## Appendices

Appendix 1: search strategy

Búsqueda pubmed

(("Lymphoma, Non-Hodgkin"[Mesh]) OR ("Lymphoma"[Mesh])) AND (("Urinary Tract"[Mesh]) OR ("Urinary Bladder"[Mesh]))

Búsqueda ovid

(exp Lymphoma, Non-Hodgkin/ OR exp Lymphoma/) AND (exp Urinary Tract/ OR exp Urinary Bladder/)

Búsqueda en Cochrane 

Search Name: Linfoma vejiga

Date Run: 13/06/2025 16:30:30

Comment: 

ID Search Hits

#1 Non Hodgkin Lymphoma 4172

#2 Lymphoma 15744

#3 Urinary Bladder 11772

#4 Urinary Tract 17518

#5 #1 OR #2 15744

#6 #3 OR #4 25384

#7 #5 AND #6 165

Búsqueda LILACS

(Linfoma no Hodgkin OR Linfoma) AND (Tracto urinario OR Vejiga urinaria)

Búsqueda EMBASE

('bladder lymphoma'/exp OR 'primary bladder lymphoma' OR 'urinary bladder lymphoma' OR ('bladder':ti,ab AND 'lymphoma':ti,ab)) 

Appendix 2: characteristics of reported cases of primary bladder lymphoma up to May 2025

**Table 2:** Characteristics of reported cases of primary bladder lymphoma. CT: Computed tomography; DA EPOCH R: Dose-adjusted EPOCH-R: etoposide, prednisone, vincristine, cyclophosphamide, doxorubicin, and rituximab; DLBCL: Diffuse large B-cell lymphoma; doxycycline, H. pylori triple therapy (clarithromycin, amoxicillin, PPI); MACOP-B: metotrexato, doxorrubicina, ciclofosfamida, vincristina, prednisona y bleomicina; MALT: mucosa-associated lymphoid tissue; MRI: magnetic resonance imaging; R-CHOP: rituximab-cyclophosphamide-doxorubicin-vincristine-prednisolone; TMP: trimethoprim; TURB: transurethral resection of bladder tumor; US: ultrasonography.

Reference	Author	Year	Country	Study type	Age	Sex	Symptom	Symptom 1	Symptom 2	Cystoscopy	Characteristics	Location	Antecedent	Background 2	Background 3	Image	Image Finding	Finding.2	High grade/Low grade	Histology (DLBCL, MZ, MALT, FL)	Bone Marrow	Stadium	QT	QT procolo	QT Cycles	Radiotherapy	Gy Radiotherapy	Radiotherapy sessions	Surgery	Surgery type	Ending	Relapse	Follow-up.
[69]	Vikas Dembla	2011	The United States	Case report	42	Male	Gross hematuria			Nodular bladder						CT	Wall thickening			Hodgkin's lymphoma	Not involved	VAT	Yes	ABVD	5	No			No		Remission	Yes	NA
[37]	J.P. Tasu	1995	France	Case series	75	Female	Gross hematuria			Single injury		Right side	Arterial hypertension	Dyslipidemia		CT	Dough		Low degree	MALT	Not involved	IAE	Yes	Cyclophosphamide		Yes			No		Remission	No	36
[37]	J.P. Tasu	1998	France	Case series	68	Male	Low back pain	Dysuria	Dysuria				Arterial hypertension	Asbestosis	Chronic cholangitis	CT	Wall thickening		High grade	DLBCL	Not involved	IAE	Yes	CHOP		No			No		Remission	Yes	5
[37]	J.P. Tasu	1995	France	Case series	70	Male	Weight loss			Normal						CT	Wall thickening		High grade	Centroblastic B lymphoma	Not involved	IBE	Yes	Mitoxantrone/Ifosfamide		No			No		Remission	No	12
[50]	Arnold B. Aigen	1986	The United States	Case report	81	Male	Gross hematuria			Single injury		Dome	Coronary heart disease	Cerebrovascular disease					Low degree	Lymphocytic lymphoma	Not involved	IAE	No			No			No		Clinical follow-up	Monitoring	36
[70]	J.S Batchelor	1991	England	Case report	65	Female	Gross hematuria	Dysuria	Frequency	Wall thickening		Later	Malacoplakia			Urogram	Filling defect			Non-Hodgkin Lymphoma		IAE	No			Yes			No		Remission	No	36
[51]	L.A Binkovitz	1988	The United States	Case report	42	Male	Gross hematuria	Low back pain	Dysuria	Single injury		Left side				CT	Wall thickening	Hydronephrosis	High grade	DLBCL	Not involved	IAE	Yes	M-BACOD		No			No		Remission	No	14
[52]	C. Álvarez Álvarez	2005	Spain	Case report	80	Female	Gross hematuria			Single injury		Right side	Cognitive disorder			CT	Wall thickening		High grade	DLBCL	Not involved	IAE	Yes	CVP		No			Yes	TURBT	Remission	No	9
[26]	Depthi Hoskoppal	2002	The United States	Case series	78	Female	Urinary tract infection			Erythematous mucosa		Right side							Low degree	MALT			Yes	Rituximab/Cyclophosphamide		No			No		Remission	Yes	60
[26]	Depthi Hoskoppal	2002	The United States	Case series	66	Male	Frequency			Single injury	Papillary		Bladder cancer						Low degree	MALT			No			No							180
[26]	Depthi Hoskoppal	2002	The United States	Case series	68	Male	Weakness	Lethargy		Trabeculation		trigone				CT	Retroperitoneal mass	Hydronephrosis	High grade	Burkitt			Yes	Cyclophosphamide/Dexamethasone		No			No		Death	No	5
[25]	Jasna Bacalja	2013	Croatia	Case report	48	Male	Incidental			Single injury		Right posterolateral				US	Wall thickening		Low degree	MALT	Not involved	IIAE	Yes	R CHOP	8	No			No		Partial Response	Partial remission	1
[53]	Jian Hui Xin	2024	China	Case report	83	Female	Frequency									US, CT	Wall asymmetry	Irregular density distal to the ureter	High grade	DLBCL			Yes	R CHOP	6	No			Yes	TURBT		It doesn't report	It doesn't report
[71]	Judella Edwinna Maria	2014	The United States	Case report	54	Male	Low back pain	Radiculopathy lose left	Left leg weakness	Single injury		Dome	Coronary heart disease	Dyslpidemia	Smoker 40 years old	CT	Dough	Wall thickening	Low degree	MALT	Not involved	IAE	No			Yes		17	Yes	TURBT	Remission		
[27]	Jui Sheng Hsu	2015	Taiwan	Case report	76	Male	Urinary tract infection			Erythematous mucosa		Right ureter orYesice				CT	Wall thickening	Hydronephrosis	Low degree	MALT	Not involved	IAE	No			Yes					Remission	It doesn't report	3
[66]	Jun Ho Choi	2003	China	Case report	30	Female	Gross hematuria	Dysuria		Erythematous mucosa	Ulcerated	Left side				Urography, US, CT	Filling defect	Lateral calcYesication		T-lymphoma	Not involved	IAE	No			No					Death		0,1
[28]	Pratima Datta Kadam	2019	Singapore, Singapore	Case report	74	Female	Frequency	Nocturia	Urinary tract infection	Wall thickening		Later				CT	Dough		Low degree	MALT	Not involved	IAE	No			Yes					Remission	It doesn't report	
[24]	S H Kashi	1990	England	Case series	57	Female	Gross hematuria			Single injury	Mobile	Left side				Urography	Filling defect		Low degree	Low-grade non-Hodgkin	Not involved	IAE	Yes	Chlorambucil/Prednisolone		No					Partial Response	Partial remission	4
[24]	S H Kashi	1990	England	Case series	74	Male	Frequency	Dysuria	Urgency	Single injury	Mobile					Urography	Filling defect		Low degree	Low-grade non-Hodgkin	Not involved	IAE	Yes	Chlorambucil		Yes	35	20			Remission	No	6
[24]	S H Kashi	1990	England	Case series	70	Male	Obstructive	Dysuria		Bulging		Later				US	Hydronephrosis		High grade	High grade non-Hodgkin	Not involved	IAE	Yes	Vincristina/Prednisolona		No					Death		1
[72]	Koji Hatano	2007	Japan	Case report	84	Female	Microscopic hematuria						Kidney tumor			CT	Dough		Low degree	MALT						Yes	36		Yes	TURBT	Remission	No	14
[8]	Marco Lucioni	2013	Italy	Case report	72	Female	Urinary tract infection	Dysuria		Wall thickening									Low degree	MALT	Not involved		Antibiotics	Ciprofloxacin		No					Remission	No	6
[54]	MD Melekos	1992	Greece	Case report	65	Male	Gross hematuria	Frequency	Urgency	Single injury		trigone				CT	Dough	Hydronephrosis	High grade	High grade non-Hodgkin	Not involved	IAE	Yes	CHOP	8	No					Remission	No	13
[55]	Nerli	2013	Indian	Case report	69	Male	Gross hematuria			Bulging		Side walls				US	Multiple injuries		High grade	High grade non-Hodgkin			Yes	R CHOP		No			Yes	TURBT	Remission	No	6
[38]	Tetsuya Imamura	2022	Japan	Case report	68	Female	Gross hematuria			Single injury	Submucosa	Left side				CT	Dough	Hydronephrosis	Low degree	MALT			Yes	Rituximab	6	Yes	30		Yes	TURBT	Remission	No	48
[33]	Michinobu Ozawa	2018	Japan	Case report	72	Male	Incidental			Bulging		Side wall							Low degree	MALT			No			No			Yes	TURBT	Remission	No	13
[56]	To Schultz	2007	Germany	Case series	69	Male	Gross hematuria						Non-Hodgkin's lymphoma						High grade	DLBCL			Yes	R CHOP	6	No			Yes	TURBT	Remission	Yes	Death
[3]	R H W Simpson	1990	The United States	Case series	70	Male	Incidental			Single injury		Dome	Bladder carcinoma						Low degree	Low grade non-Hodgkin	Not involved	IIAE	No			Yes			Yes	Cystectomy	Remission	No	84
[3]	R H W Simpson	1990	The United States	Case series	67	Female	Gross hematuria			Single injury	Sesil	Right side							High grade	DLBCL									Yes	Partial cystectomy	Remission	Yes	36
[23]	Tatjana Terzic	2008	Serbia	Case report	54	Female	Dysuria	Night sweats		Mucous congestion						CT	Dough		Low degree	Lymphocytic lymphoma	Not involved	IBE	Yes	LOP	6	No					Remission	Yes	108
[9]	J van den Bosch	2002	Netherlands	Case report	59	Male	Gross hematuria			Erythematous mucosa									Low degree	MALT			Antibiotics	H pylori		No					Remission	No	36
[65]	Yang Ke-xun	2021	China	Case report	81	Female	Incontinence	Dysuria		Single injury	Ulcerated									T NK Lymphoma									Yes	TURBT	Death		2
[57]	L J Yeoman	1991	England	Case series	32	Female	Dysuria	Urgency		Single injury	Nodular					US	Hydronephrosis		High grade	High grade non-Hodgkin			Yes	MACOP-B							Remission	It doesn't report	It doesn't report
[22]	Nina Combaz	2017	Swiss	Case report	67	Male	Urinary tract infection	Incontinence	Gross hematuria	Normal						US, CT	No injuries		Low degree	MALT			Yes	RItuximab		No					Remission	No	36
[21]	M. J. Fernadez Aceñero	1996	Spain	Case series	73	Female	Low back pain	Dysuria								CT	Dough		High grade	High grade non-Hodgkin			Yes	It doesn't report							Remission	No	8
[21]	M. J. Fernadez Aceñero	1996	Spain	Case series	50	Female	Fever									CT	Dough		Low degree	Low grade non-Hodgkin			Yes	It doesn't report							Remission	No	69
[21]	M. J. Fernadez Aceñero	1996	Spain	Case series	75	Female	Dysuria						Breast cancer			CT	Dough		High grade	High grade non-Hodgkin			Yes	It doesn't report							Remission	No	69
[29]	Jauah Al Maghrabi	2000	Canada.	Case series	64	Female	Gross hematuria	Frequency	Urinary tract infection	Single injury	Ulcerated								Low degree	Lymphocytic lymphoma	Not involved	IAE				Yes	35	20			Remission	No	136
[29]	Jauah Al Maghrabi	2000	Canada.	Case series	69	Female	Frequency	Urgency	Urinary tract infection	Multiple injuries	Nodular					CT	Hydronephrosis	Wall thickening	Low degree	MALT	Not involved	IAE				Yes	35	20			Remission	No	60
[29]	Jauah Al Maghrabi	2000	Canada.	Case series	72	Female	Microscopic hematuria	Nocturia	Urinary tract infection										Low degree	MALT	Not involved	IAE				Yes	35	20			Remission	No	36
[29]	Jauah Al Maghrabi	2000	Canada.	Case series	62	Male	Gross hematuria	Frequency													Not involved	IAE				Yes	35	20			Remission	No	24
[34]	Ko Ando	1999	Japan	Case report	77	Female	Obstructive	Dysuria		Single injury	Submucosa	Left side							Low degree	MALT			No			No	No		Yes	TURBT	Remission	No	36
[44]	Atsuto Katano	2021	Japan	Case report	65	Female	Gross hematuria	Urinary tract infection		Single injury	Submucosa	trigone				MRI	Dough		Low degree	MALT	Not involved	IAE				Yes	30	15	Yes	TURBT	Remission	No	24
[49]	A W Bates	2000	England	Case series	66	Female	Incidental												Low degree	MALT		IE									Remission		
[49]	A W Bates	2000	England	Case series	79	Female	Gross hematuria			Multiple injuries									Low degree	MALT		IE											
[49]	A W Bates	2000	England	Case series	59	Female	He doesn't describe			Single injury	Submucosa	Previous wall							Low degree	MALT											Clinical follow-up		36
[49]	A W Bates	2000	England	Case series	84	Female	Gross hematuria			Mucous congestion		Right ureter orYesice							High grade	DLBCL		IE									Death		6
[49]	A W Bates	2000	England	Case series	67	Male	Gross hematuria			Single injury	Irregular	Right side							High grade	DLBCL		IE	Yes			Yes					Remission	No	192
[49]	A W Bates	2000	England	Case series	80	Female	Gross hematuria			Single injury		Right ureter orYesice							High grade	DLBCL	Not involved	IAE				Yes					Remission		44
[45]	Jerry Lorren Dominic	2024	The Ukraine	Case report	69	Female	Gross hematuria									CT	Dough	Hydronephrosis	Low degree	MALT vs Follicular	Not involved	IAE				Yes	30	15	Yes	TURBT	Remission		
[10].	Fujimura	2008	Japan	Case report	69	Male	Gross hematuria			Erythematous mucosa		trigone				CT	Wall thickening		Low degree	MALT		IAE	Antibiotics	H pylori					Yes	TURBT	Remission		25
[11]	Marie Hughes	2005	United Kingdom	Case series	82	Female	Gross hematuria												Low degree	MALT			Yes	Chlorambucil, Vincristine, Prednisolone							Death		
[11]	Marie Hughes	2005	United Kingdom	Case series	87	Female	Urinary tract infection												Low degree	Low grade non-Hodgkin			Antibiotics	Doxycycline							Remission		
[11]	Marie Hughes	2005	United Kingdom	Case series	75	Female	Gross hematuria												High grade	High grade non-Hodgkin			Yes	Doxorubicin, Chlorambucil, Prednisolone							Death		
[11]	Marie Hughes	2005	United Kingdom	Case series	81	Female	Gross hematuria												Low degree	MALT									Yes	Diathermia	Remission	No	12
[11]	Marie Hughes	2005	United Kingdom	Case series	27	Male	Gross hematuria	Pain in the iliac fossa											High grade	Anaplastic lymphoma			Yes	CHOP / Chlorambucil, Vincristine, Prednisolone x 6	4/6				Yes	Surgical resection	Remission	No	84
[11]	Marie Hughes	2005	United Kingdom	Case series	28	Male	Gross hematuria												Low degree	MALT			Yes	Chlorambucil, Vincristine, Prednisolone	6						Remission	No	120
[11]	Marie Hughes	2005	United Kingdom	Case series	76	Female	Gross hematuria												Low degree	MALT						Yes	35	20			Remission	No	24
[11]	Marie Hughes	2005	United Kingdom	Case series	77	Male	Gross hematuria												Low degree	MALT			Yes	Chlorambucil, Idoxurubicin, Dexamethasone							Remission	No	48
[11]	Marie Hughes	2005	United Kingdom	Case series	71	Female	Gross hematuria												High grade	DLBCL			Yes	CHOP	6						Remission	No	24
[11]	Marie Hughes	2005	United Kingdom	Case series	31	Male	Urinary tract infection												High grade	DLBCL			Yes	Doxycycline							Remission	No	96
[11]	Marie Hughes	2005	United Kingdom	Case series	70	Female	Gross hematuria												High grade	DLBCL			Yes	CHOP	6				Yes	Surgical resection	Remission	No	48
[11]	Marie Hughes	2005	United Kingdom	Case series	66	Female	Urinary tract infection												Low degree	MALT											Death		
[20]	Kempton Christie L.	1997	The United States	Case series	52	Male													Low degree	MALT		IIIA				Yes					Death		36
[20]	Kempton Christie L.	1997	The United States	Case series	46	Female													Low degree	Follicular		IIAE	Yes			Yes			Yes	Upper vaginectomy,	Remission	No	132
[20]	Kempton Christie L.	1997	The United States	Case series	68	Female													Low degree	Follicular		VAT	Yes			Yes			Yes	Fulguration	Remission	No	12
[20]	Kempton Christie L.	1997	The United States	Case series	62	Female													Low degree	Follicular		VAT				Yes							
[20]	Kempton Christie L.	1997	The United States	Case series	48	Male													Low degree	Follicular		VAT				Yes			Yes	TURBT	Death		108
[20]	Kempton Christie L.	1997	The United States	Case series	42	Male													Low degree	Follicular		VAT	Yes								Remission	No	180
[20]	Kempton Christie L.	1997	The United States	Case series	32	Male													High grade	DLBCL						Yes					Remission		24
[20]	Kempton Christie L.	1997	The United States	Case series	28	Male													High grade	DLBCL						Yes							
[20]	Kempton Christie L.	1997	The United States	Case series	72	Male													High grade	DLBCL		IIA	Yes			Yes					Death		60
[20]	Kempton Christie L.	1997	The United States	Case series	54	Male													High grade	DLBCL		IIA				Yes					Death		4
[20]	Kempton Christie L.	1997	The United States	Case series	5	Male													High grade	DLBCL		VAT									Death		0
[20]	Kempton Christie L.	1997	The United States	Case series	57	Female													High grade	DLBCL		VAT				Yes					Remission		36
[20]	Kempton Christie L.	1997	The United States	Case series	60	Male													High grade	DLBCL		IVB	Yes						Yes	Cystectomy	Death		3
[20]	Kempton Christie L.	1997	The United States	Case series	45	Female													High grade	DLBCL		IBE				Yes					Remission		120
[20]	Kempton Christie L.	1997	The United States	Case series	63	Female													High grade	DLBCL						Yes					Remission		120
[20]	Kempton Christie L.	1997	The United States	Case series	61	Female													High grade	DLBCL									Yes	Partial cystectomy	Remission		96
[20]	Kempton Christie L.	1997	The United States	Case series	66	Female													High grade	DLBCL											Death		0
[20]	Kempton Christie L.	1997	The United States	Case series	50	Female													Low degree	MALT		IAE				Yes			Yes	Surgical resection	Remission		240
[20]	Kempton Christie L.	1997	The United States	Case series	64	Female													Low degree	MALT		IAE							Yes	Partial cystectomy	Remission		144
[20]	Kempton Christie L.	1997	The United States	Case series	73	Female													Low degree	MALT		IAE				Yes			Yes	Fulguration	Remission		120
[20]	Kempton Christie L.	1997	The United States	Case series	79	Female													Low degree	MALT		IAE				Yes					Remission	No	24
[20]	Kempton Christie L.	1997	The United States	Case series	27	Female													Low degree	MALT		IAE				Yes					Remission		480
[20]	Kempton Christie L.	1997	The United States	Case series	45	Female													Low degree	MALT		IAE				Yes					Remission		312
[12].	S M Kröber	2002	Germany	Case report	57	Male	Dysuria	Obstructive		Single injury		Roof	MALT stomach						Low degree	MALT	Not involved	IAE	Antibiotics	H pylori					Yes	TURBT	Remission		36
[73]	Katiar Leite	2004	Brazil	Case report	89	Female	Obstructive			Wall thickening		Right side	History of breast ductal carcinoma, relapsed in the skin			MRI	Wall thickening						Yes	CHOP	6				Yes	TURBT	Remission	Yes	12
[46]	Po Sung Liang	2024	Taiwan	Case report	79	Female	Urgency	Urinary tract infection	Frequency				Arterial hypertension	Diabetes mellitus		Ultrasound, CT	Dough		Low degree	MALT	Not involved	IAE				Yes	24	20	Yes	TURBT	Remission		9
[74]	Lucien Bory Porras	2023	Cuba	Case report	58	Male	Gross hematuria	Obstructive	Weight loss	Wall thickening		Right side				US	Dough		High grade	High grade non-Hodgkin	Not involved	IAE	Yes	CHOP	6	Yes			Yes	TURBT	Remission	No	44
[39]	K G Maninderpal	2011	Malaysia	Case report	65	Female	Nausea	Vomit	Dizziness	Wall thickening			Diabetes	Anemia		US, CT	Wall thickening	Hydronephrosis	Low degree	MALT	Infiltrator	IVE	Yes	R CHOP					Yes	TURBT	Remission	Yes	3
[30]	Akinori Masuda	2002	Japan	Case report	56	Male	Gross hematuria			Single injury		Previous							Low degree	MALT						Yes	50				Remission		21
[19]	Ikuo Matsuda	2014	Japan	Case report	78	Female	Urinary tract infection									MRI, CT	Dough	Hydronephrosis	Low degree	MALT			Yes	Rituximab	4						Partial Response		
[59]	Joseph Alburqueque	2024	Peru.	Case report	83	Female	Gross hematuria			Single injury	Ulcerated		Arterial hypertension	Glaucoma		CT	Dough	Hydronephrosis	High grade	DLBCL			Yes	R CHOP	6				Yes	TURBT	Remission		
[40]	Kei Mizuno	2013	Japan	Case report	72	Female	Urinary tract infection	Microscopic hematuria		Single injury	Submucosa	Right side				CT	Dough	Hydronephrosis	Low degree	MALT	Not involved	IAE	Yes	Rituximab		Yes			Yes	TURBT	Remission		
[18]	Ken Morita	2012	Japan	Case report	68	Female	Urinary tract infection	Suprapubic pain	Frequency	Single injury	Ulcerated								Low degree	MALT		IE	Yes	Rituximab	4						Remission		
[14]	D Oscier	2002	England	Case report	78	Female	Urinary tract infection			Single injury		Left side							Low degree	MALT	Not involved	IAE	Antibiotics	Trimethoprim, nitrofurantoin, and cephradine							Remission	No	19
[58]	Baijaeek Sain	2023	England	Case report	80	Female	Abdominal pain	Constrint	Weight loss				Transverse myelitis	Hypertension		MRI, CT	Wall thickening	Hydronephrosis	High grade	DLBCL, EBV positive									Yes	TURBT catheter double J	Remission		16
[60]	Hideshige Seki	2024	Japan	Case report	77	Male	Fever	Discomfort								CT	Wall thickening	Dough	High grade	DLBCL		IE	Yes	R CHOP	3						Progression/Palliative treatment		
[17]	S Sen	2010	England	Case report	31	Female	Fever	Gross hematuria					Secondary epilepsy	CV malformation		US	Dough		Low degree	MALT	Not involved		Yes	R COP									
[3]	W Greg Simpson	2014	The United States	Case report	48	Male	Gross hematuria	Low back pain	Frequency							CT	Dough	Hydronephrosis	High grade	DLBCL	Not involved	IAE	Yes	R CHOP	3				Yes	TURBT	Remission	No	6
[75]	Tomaz R Szopinski	2010	Poland	Case report	17	Female				Single injury	Nodular		Arterial hypertension			US	Dough		Low degree	MALT	Not involved		Yes	CVP					Yes	TURBT	Remission	No	24
[35]	K Nakanishi	2012	Japan	Case report	71	Female	Gross hematuria									MRI	Dough		Low degree	MALT									Yes	TURBT			
[13]	Tadashi Terada	2011	Japan	Case report	88	Female	Gross hematuria			Single injury	Polypoid	Later							Low degree	MALT			Antibiotics						Yes	TURBT	Remission		6
[47]	Prashant Vempati	2015	The United States	Case report	65	Female	Gross hematuria			Single injury		Right side							Low degree	MALT						Yes	30		Yes	TURBT	Remission		3
[76]	Hassan D Wazait	2000	England	Case series	70	Female	Gross hematuria			Single injury		Left ureter orYesice							Low degree	MALT									Yes	TURBT	Remission	Yes	12
[76]	Hassan D Wazait	2000	England	Case series	65	Female	Gross hematuria	Dysuria	Frequency	Mucous congestion									Low degree	MALT		IAE	Yes	CHOP							Remission	No	36
[4]	Xi you	2023	The United States	Case report	81	Female	Frequency	Dysuria								US	Dough		Low degree	MALT									Yes	TURBT	Remission		12
[36]	Hewei Xu	2020	China	Case report	77	Female	Frequency	Urgency	Dysuria	Occupied by a tumor						CT	Nodular wall		Low degree	MALT	Not involved	IAE							Yes	TURBT	Remission		24
[61]	Yi Wang	2016	China	Case report	62	Male	Gross hematuria	Frequency	Dysuria							CT	Dough		High grade	DLBCL			Yes	R CHOP	6						Remission	No	16
[15]	Yoko Ueno	2007	Japan	Case report	64	Female	Incidental			Single injury	Submucosa		Ovarian tumor						Low degree	MALT						Yes	40		Yes	TURBT	Remission	Yes	9
[62]	Samuel K Luketich	2023	The United States	Case report	66	Male	Gross hematuria	Low back pain		Single injury				Multiple myeloma		CT	Dough		High grade	DLBCL		VAT	Yes	R CHOP					No	TURBT	It doesn't report		
[63]	Soum Lokeshwar	2021	The United States	Case report	57	Male	Dysuria	Frequency	Microscopic hematuria	Single injury						CT	Wall thickening		High grade	DLBCL	Infiltrator	VAT	Yes	DA EPOCH R					No		Progression		
[64]	Akinori Hayashi	2009	Japan	Case report	75	Female	Microscopic hematuria									CT	Wall thickening	Hydronephrosis	High grade	DLBCL	Not involved	VAT	Yes	R CHOP	3				No		Remission		
[31]	J Pawade	1993	England	Case series	67	Female	Incontinence	Dysuria	Urinary tract infection										Low degree	MALT									Yes	Cystectomy	Remission		24
[31]	J Pawade	1993	England	Case series	74	Female	Dysuria			Dough									Low degree	MALT						Yes					Death		
[31]	J Pawade	1993	England	Case series	22	Female	Abdominal pain	Gross hematuria	Dysuria	Dough		Right side							Low degree	MALT			Yes			Yes					Remission	No	46
[31]	J Pawade	1993	England	Case series	83	Female													Low degree	MALT						Yes					Remission		20
[31]	J Pawade	1993	England	Case series	80	Male													Low degree	MALT											Clinical follow-up		20
[41]	Hajime Kuhara	1990	Japan	Case report	56	Female	Frequency	Urinary tract infection		Multiple injuries						CT	Wall thickening		Low degree	MALT	Not involved		Yes						Yes	Cystectomy	Remission	No	9
[48]	F Yuille	1998	England	Case report	80	FEMALE	Gross hematuria			Dough		Later				CT	Wall thickening		Low degree	MALT	Not involved					Yes	40	20	Yes	TURBT	Remission		16
[42]	Kirill A Lyapichev	2021	The United States	Case report	58	FEMALE	Frequency	Dysuria		Dough		Later	Arterial hypertension	Rheumatoid arthritis		CT	Wall thickening		Low degree	MALT			Yes	Rituximab					Yes	TURBT	Remission	No	120
[16]	Yoichi Kakuta	2006	Japan	Case report	84	MALE	Weight loss									CT	Dough		Low degree	MALT			Yes	R CHOP	1						Remission		
[32]	Makoto Isono	2018	Japan	Case report	77	FEMALE	Frequency	Gross hematuria	Urinary tract infection	Erythematous mucosa		Later				MRI	Dough		Low degree	MALT						Yes	30.6	17			Remission		60
[43]	Ankti Jitani	2016	Indian	Case report	53	FEMALE	Urgency	Dysuria	Urinary tract infection							CT	Wall thickening		Low degree	MALT	Not involved	IAE	Yes	CHOP	6				Yes	TURBT	Remission	No	9

## References

[1] Shankland K, Armitage J, Hancock B (2012). Non-Hodgkin lymphoma. Lancet Lond Engl.

[2] Thandra KC, Barsouk A, Saginala K, Padala SA, Barsouk A, Rawla P (2021). Epidemiology of non-Hodgkin's lymphoma. Med Sci (Basel).

[3] Simpson WG, Lopez A, Babbar P, Payne LF (2015). Primary bladder lymphoma, diffuse large B-cell type: case report and literature review of 26 cases. Urol Ann.

[4] Tu X, Zhuang X, Li F, Huang C, Qian Y (2022). Rare primary bladder mucosa-associated lymphoid tissue lymphoma: a case report and review of literature. Front Oncol.

[5] Zanelli M, Sanguedolce F, Zizzo M (2022). Primary diffuse large B-cell lymphoma of the urinary bladder: update on a rare disease and potential diagnostic pitfalls. Curr Oncol.

[6] Lontos K, Tsagianni A, Msaouel P, Appleman LJ, Nasioudis D (2017). Primary urinary tract lymphoma: rare but aggressive. Anticancer Res.

[7] Venyo AK (2014). Lymphoma of the urinary bladder. Adv Urol.

[8] Lucioni M, Nicola M, Riboni R (2013). Antibiotic therapy-induced remission of bladder mucosa-associated lymphoid tissue (MALT) lymphoma carrying t(11;18)(q21;q21) apoptosis inhibitor 2-MALT1. J Clin Oncol.

[9] van den Bosch J, Kropman RF, Blok P, Wijermans PW (2002). Disappearance of a mucosa-associated lymphoid tissue (MALT) lymphoma of the urinary bladder after treatment for Helicobacter pylori. Eur J Haematol.

[10] Fujimura M, Chin K, Sekita N (2008). Regression of mucosa-associated lymphoid tissue lymphoma of the bladder after antibiotic therapy: a case report (Article in Japanese). Hinyokika Kiyo.

[11] Hughes M, Morrison A, Jackson R (2005). Primary bladder lymphoma: management and outcome of 12 patients with a review of the literature. Leuk Lymphoma.

[12] Kröber SM, Aepinus C, Ruck P, Müller-Hermelink HK, Horny HP, Kaiserling E (2002). Extranodal marginal zone B cell lymphoma of MALT type involving the mucosa of both the urinary bladder and stomach. J Clin Pathol.

[13] Terada T (2011). Primary CD5-positive mucosa-associated lymphoid tissue lymphoma of the urinary bladder. Ann Diagn Pathol.

[14] Oscier D, Bramble J, Hodges E, Wright D (2002). Regression of mucosa-associated lymphoid tissue lymphoma of the bladder after antibiotic therapy. J Clin Oncol.

[15] Ueno Y, Sakai H, Tsuruta T, Masahisa W (2007). Mucosa-associated lymphoma of the bladder with relapse in the stomach after successful local treatment (Article in Japanese). Hinyokika Kiyo.

[16] Kakuta Y, Katoh T, Saitoh J, Yazawa K, Hosomi M, Itoh K (2006). A case of primary mucosa-associated lymphoid tissue lymphoma of the bladder regressed after rituximab in combination with CHOP chemotherapy (Article in Japanese). Hinyokika Kiyo.

[17] Sen S, Macaulay JH, Allford SL (2010). A case of cerebral arteriovenous malformation in pregnancy associated with MALT lymphoma. J Obstet Gynaecol.

[18] Morita K, Nakamura F, Nannya Y (2012). Primary MALT lymphoma of the urinary bladder in the background of interstitial cystitis. Ann Hematol.

[19] Matsuda I, Zozumi M, Tsuchida YA (2014). Primary extranodal marginal zone lymphoma of mucosa-associated lymphoid tissue type with malakoplakia in the urinary bladder: a case report. Int J Clin Exp Pathol.

[20] Kempton CL, Kurtin PJ, Inwards DJ, Wollan P, Bostwick DG (1997). Malignant lymphoma of the bladder: evidence from 36 cases that low-grade lymphoma of the MALT-type is the most common primary bladder lymphoma. Am J Surg Pathol.

[21] Acenero MJ, Rodilla CM, García-Asenjo JL, Menchero SC, Esponera JS (1996). Primary malignant lymphoma of the bladder. Pathol Res Pract.

[22] Combaz N, Kuhn A (2017). Case report about a primary bladder lymphoma. Int Arch Urol Complicat.

[23] Terzic T, Radojevic S, Cemerikic-Martinovic V (2008). Primary non-hodgkin lymphoma of urinary bladder with nine years later renal involvement and absence of systemic lymphoma: a case report. Med Oncol.

[24] Kashi SH, Murphy JK, Britton JP, Whelan P (1990). Primary lymphoma of the bladder: a clinicopathological study of 3 cases. Eur Urol.

[25] Bacalja J, Ulamec M, Rako D, Bošković L, Trnski D, Vrdoljak E, Krušlin B (2013). Persistence of primary MALT lymphoma of the urinary bladder after rituximab with CHOP chemotherapy and radiotherapy. In Vivo.

[26] Hoskoppal D, Ren Q, Huang H, Park K, Deng FM (2022). Malignant lymphoma of the lower urinary tract: a single institutional experience. Pathol Res Pract.

[27] Hsu JS, Lin CC, Chen YT, Lee YC (2015). Primary mucosa-associated lymphoid tissue lymphoma of the urinary bladder. Kaohsiung J Med Sci.

[28] Kadam PD, Han HC, Kwok JL (2019). An uncommon case of mucosa-associated lymphoid tissue (MALT) tumor of the bladder. Int Urogynecol J.

[29] Al-Maghrabi J, Kamel-Reid S, Jewett M, Gospodarowicz M, Wells W, Banerjee D (2001). Primary low-grade B-cell lymphoma of mucosa-associated lymphoid tissue type arising in the urinary bladder: report of 4 cases with molecular genetic analysis. Arch Pathol Lab Med.

[30] Masuda A, Tsujii T, Kojima M, Sakamoto S, Moriguchi H, Honda M, Yoshida K (2002). Primary mucosa-associated lymphoid tissue (MALT) lymphoma arising from the male urethra. A case report and review of the literature. Pathol Res Pract.

[31] Pawade J, Banerjee SS, Harris M, Isaacson P, Wright D (1993). Lymphomas of mucosa-associated lymphoid tissue arising in the urinary bladder. Histopathology.

[32] Isono M, Sato A, Kimura F, Asano T (2018). A case of mucosa-associated lymphoid tissue lymphoma of the bladder successfully treated with radiotherapy. Urol Case Rep.

[33] Ozawa M, Suenaga S, Ishii T, Suzuki H, Tsuchiya N, Ohtake H (2018). Primary malignant lymphoma of the bladder diagnosed by transurethral bladder tumor resection: a case report (Article in Japanese). Nihon Hinyokika Gakkai Zasshi.

[34] Ando K, Matsuno Y, Kanai Y, Sakamoto M, Fujimoto H, Narabayashi M, Tobisu K (1999). Primary low-grade lymphoma of mucosa-associated lymphoid tissue of the urinary bladder: a case report with special reference to the use of ancillary diagnostic studies. Jpn J Clin Oncol.

[35] Takahashi H, Shimazaki H, Oda T (2013). Malignant lymphoma case with urinary cytology mimicking that of urothelial carcinoma. Cytopathology.

[36] Xu H, Chen Z, Shen B, Wei Z (2020). Primary bladder mucosa-associated lymphoid tissue lymphoma: a case report and literature review. Medicine (Baltimore).

[37] Tasu JP, Geffroy D, Rocher L (2000). Primary malignant lymphoma of the urinary bladder: report of three cases and review of the literature. Eur Radiol.

[38] Imamura T, Miyachi S, Horiuchi E, Ikeda T (2022). A case of primary mucosa-associated lymphoid tissue (MALT) lymphoma of the urinary bladder (Article in Japanese). Nihon Hinyokika Gakkai Zasshi.

[39] Maninderpal KG, Amir FH, Azad HA, Mun KS (2011). Imaging findings of a primary bladder maltoma. Br J Radiol.

[40] Mizuno K, Nakanishi S, Sakatani T (2013). A case of primary mucosa-associated lymphoid tissue-type lymphoma of the urinary bladder that progressed after antibiotic therapy (Article in Japanese). Hinyokika Kiyo.

[41] Kuhara H, Tamura Z, Suchi T, Hattori R, Kinukawa T (1990). Primary malignant lymphoma of the urinary bladder. A case report. Acta Pathol Jpn.

[42] Lyapichev KA, Ivashkevich Y, Chernov Y (2021). MALT lymphoma of the urinary bladder shows a dramatic female predominance, uneven geographic distribution, and possible infectious etiology. Res Rep Urol.

[43] Jitani AK, Mishra J, Sailo SL, Raphael V (2016). Primary urinary bladder mucosa associated lymphoid tissue type lymphoma presenting as a close mimic for genitourinary tuberculosis: case report and review of literature. Urol Ann.

[44] Katano A, Yamashita H (2022). Primary urinary bladder marginal zone B-cell lymphoma of mucosa-associated lymphoid tissue. J Cancer Res Ther.

[45] Dominic JL, Ganduboina R, Dutta P, Gubran K, Toussaint ML, Isrow DM (2024). Primary bladder B-cell lymphoma: a rare case report and review of literature. Ann Med Surg (Lond).

[46] Liang PS, Shih HJ, Huang SH, Chen YZ (2024). Primary lymphoma of mucosa associated lymphoid tissue (MALT lymphoma) in the urinary bladder mimicking recurrent urinary tract infection: a case report and literature review. BMC Urol.

[47] Vempati P, Knoll MA, Alqatari M, Strauchen J, Malone AK, Bakst RL (2015). MALT lymphoma of the bladder: a case report and review of the literature. Case Rep Hematol.

[48] Yuille FA, Angus B, Roberts JT, Vadanan BS (1998). Low-grade MALT lymphoma of the urinary bladder. Clin Oncol (R Coll Radiol).

[49] Bates AW, Norton AJ, Baithun SI (2000). Malignant lymphoma of the urinary bladder: a clinicopathological study of 11 cases. J Clin Pathol.

[50] Aigen AB, Phillips M (1986). Primary malignant lymphoma of urinary bladder. Urology.

[51] Binkovitz LA, Hattery RR, LeRoy AJ (1988). Primary lymphoma of the bladder. Urol Radiol.

[52] Álvarez A, Pérez-Quintela BV, Santiago DP, Armentia ES, Fraile PS, Badiola IA (2005). Primary large cell non-Hodgkin B lymphoma of the bladder. Presenting a Case (Article in Spanish). Actas Urol Esp.

[53] Xin JH, Jiang B, Yuan YH, Zou XF (2024). Primary diffuse large B-cell lymphoma of the urinary bladder: a case report. Asian J Surg.

[54] Melekos MD, Matsouka P, Fokaefs E, Pantazakos A, Repanti M (1992). Primary non-Hodgkin's lymphoma of the urinary bladder. Eur Urol.

[55] Nerli RB, Guntaka AK, Das S, Hiremath MB (2013). Primary non-Hodgkin lymphoma of the bladder. Indian J Cancer.

[56] Schultz A, Maruschke M, Leithäuser M, Seiter H (2007). Genitourinary manifestations of lymphoma (Article in German). Aktuelle Urol.

[57] Yeoman LJ, Mason MD, Olliff JF (1991). Non-Hodgkin’s lymphoma of the bladder — CT and MRI appearances. Clin Radiol.

[58] Sain B, Blake M, Goyal K, Kaur H, Robinson K (2023). Epstein-Barr virus-positive primary diffuse large B-cell lymphoma of the urinary bladder: a case report. J Surg Case Rep.

[59] Alburqueque-Melgarejo J, Garate BB (2024). Primary urinary bladder lymphoma: presentation with bilateral hydronephrosis. Hematol Clin Pract.

[60] Seki H, Mizuno S, Saigusa S (2024). Primary bladder lymphoma with extravesical extension: a case report and literature review on prognosis and clinical characteristics. J Clin Med.

[61] Wang Y, Xing Q, Guo Z (2016). Primary diffuse large B-cell lymphoma of the urinary bladder: a case report and review of literature. Int J Clin Exp Pathol.

[62] Luketich SK, Zekan DS, Prisneac I, Howell DM, El Naili R, Zaslau S (2023). EBV-positive diffuse large B-cell lymphoma presenting as symptomatic masses in the bladder trigone and unilateral kidney. Urol Case Rep.

[63] Lokeshwar SD, Rahman SN, Syed J, Monaghan TF, Press B, Soloway MS (2022). Diffuse large B-cell lymphoma presenting as LUTS: clinical practice points. Urol Case Rep.

[64] Hayashi A, Miyakawa Y, Bokuda K (2009). Primary diffuse large B-cell lymphoma of the bladder. Intern Med.

[65] Ke-Xun Y, Qin-Zhang W (2022). An uncommon case of NK/T-cell lymphoma with involvement of the urinary bladder. Int Urogynecol J.

[66] Choi JH, Jeong YY, Shin SS, Lim HS, Kang HK (2003). Primary calcified T-cell lymphoma of the urinary bladder: a case report. Korean J Radiol.

[67] Tsang RW, Gospodarowicz MK, Pintilie M (2001). Stage I and II MALT lymphoma: results of treatment with radiotherapy. Int J Radiat Oncol.

[68] Dembla V, Walker BN, Elkins SL, Files JC (2011). Hematuria—a rare presentation of Hodgkin lymphoma. Clin Adv Hematol Oncol.

[69] Batchelor JS, Philp NH, Ramsden KL, Scott KW (1991). Primary lymphoma of the bladder arising from an area of Malakoplakia. Br J Urol.

[70] Haddad-Lacle JE, Haddad CJ, Villas B (2014). A rare urinary bladder tumour. BMJ Case Rep.

[71] Hatano K, Sato M, Tsujimoto Y (2007). Primary mucosa-associated lymphoid tissue (MALT) lymphoma of the urinary bladder associated with left renal pelvic carcinoma: a case report (Article in Japanese). Hinyokika Kiyo.

[72] Leite KR, Bruschini H, Camara-Lopes LH (2004). Primary lymphoma of the bladder. Int Braz J Urol.

[73] Porras LB, Silva JW, Valle YF, Garcia DM, Rodríguez MO, Benet RO (2024). Bladder lymphoma. Presentation of clinical case. Multidiscip Montev.

[74] Szopiński TR, Sudoł-Szopińska I, Dzik T, Borówka A, Dembowska-Bagińska B, Perek D (2011). Incidental sonographic detection of mucosa-associated lymphoid tissue lymphoma of the urinary bladder found in a very young woman: report of a case. J Clin Ultrasound.

[75] Wazait HD, Chahal R, Sundurum SK, Rajkumar GN, Wright D, Aslam MM (2001). MALT-type primary lymphoma of the urinary bladder: clinicopathological study of 2 cases and review of the literature. Urol Int.

